# Quality of Life in Vietnamese Gastric Cancer Patients

**DOI:** 10.1155/2019/7167065

**Published:** 2019-05-19

**Authors:** Dzung Ngoc Thi Dang, Lan Ngoc Thi Nguyen, Nga Thi Dang, Huy Quang Dang, Thanh Van Ta

**Affiliations:** ^1^Biochemistry Department, Hanoi Medical University, Hanoi, Vietnam; ^2^Department of Medical Laboratory Science, Faculty of Medical Technology, Hanoi Medical University, Hanoi, Vietnam

## Abstract

**Background:**

Gastric cancer (GC) is one of the leading causes of cancer-related death in Vietnam. Research on health-related quality of life of Vietnamese gastric cancer patients is still in its infancy.

**Aim:**

To assess the health-related quality of life (HRQOL) of GC patients using the 15D instrument.

**Materials and Method:**

182 Vietnamese gastric cancer patients were selected to be interviewed and their HRQOL was assessed using the generic 15D questionnaire. Tables regarding history, disease characteristics, and HRQOL of participants were formulated according to genders using STATA 12.0.

**Results:**

The average age of the participants was 60.8 ± 11.6. The average time from diagnosis to the date of interview was 14.8 ± 8.4 months. The health-related quality of life (HRQOL) index score of gastric cancer patients using the 15D instrument was 0.92 ± 0.08, in which the “sexual activity” dimension had the lowest score of 0.66. Also, our study found several factors affecting HRQOL, including age, occupation, education, disease stage, treatment, and time from the date of diagnosis.

**Conclusion:**

The 15D instrument was a suitable tool to assess Vietnamese gastric cancer patients' quality of life. Findings from the study suggest the importance of frequently measuring personal functioning and performance of GC patients as parts of QOL assessment during clinical examination. It also implies the needs for more focused policies on raising the overall quality of life of patients such as encouragement of periodical HQROL assessment and acknowledging HRQOL as a treatment/intervention goal besides the 5-year survival rate.

## 1. Introduction

Being the third leading cause of cancer death and the fifth most common cancer worldwide, gastric cancer (GC) can be considered one of the world's biggest medical challenges [1]. In 2018, more than a million new cases of GC and about 783 thousand deaths have been reported by GLOBOCAN, most of which occurs in East Asia with the age-standardized incidence rate (ASR) of 32.1 per 100,000 for males and 13.2 per 100,000 for females [1]. In Vietnam, the ASR has been reported to be about 16.3 per 100,000 people of both sexes, which was the highest rate compared to other South-East Asian countries [2]. The mortality rates of GC for both males and females were also among the highest of cancers in Vietnam, surpassed only by liver cancer and lung cancer [3]. This has been argued to be attributable to the high prevalence of* Helicobacter pylori* (as much as 75% in Vietnamese adults), in combination with other risk factors such as smoking, obesity, and socioeconomic status [4, 5]. It has been suggested in a recent study that understanding GC patients' health-related quality of life (HRQOL) would be of great importance to optimize treatment plans and raise awareness among patients and health practitioners [6]. However, general research on GC in Vietnam has mostly focused on disease management and treatment to increase the 5-year survival rate, creating a gap in the HRQOL of GC patients.

The purpose of this study was to assess the HRQOL of Vietnamese GC patients using the 15D instrument and by doing so determining aspects that can be improved for the overall enhancement of healthcare for the patients. Findings regarding factors that affect patients' quality of life based on Vietnamese socioeconomic characteristics may also provide directions for healthcare policy improvements.

## 2. Materials and Methods

### 2.1. Study Design

A cross-sectional study was conducted from April to September 2018 in four hospitals in Hanoi, Vietnam, namely, National Cancer Hospital, Hanoi Medical University Hospital, 108 Military Central Hospital, and Viet Duc Hospital. These have been considered among the largest and most populated hospitals in the North of Vietnam, which housed a rather diverse patient population, many being transferred from lower level health facilities (provincial, local, etc.).

Participant selection criteria include (1) being diagnosed with gastric cancer (medical records with histopathology confirmation); (2) agreeing to participate in the study; (3) being able to communicate with the interviewers via telephone. Medical records from participants were collected under the agreement of the administrative departments of the four hospitals. We excluded participants who suffered from serious illnesses that might hinder the interview process; such illnesses included deafness, muteness, or in comatose state. Information including name, age, addresses, and phone numbers of 310 patients diagnosed with GC was gathered using medical records from hospitals. There were 30 patients who died before being contacted for participation and 98 patients could not be contacted; this resulted in 182 gastric cancer patients participating in the study. The study proposal, which included a method section covering patients' consents, was approved by the Institutional Review Board. During the process of obtaining patients' consent, we had approached patients using information from the four hospitals and made sure the patients understand about the following: the research's purpose and duration, basic procedure, foreseeable risks and discomforts, benefits, and statement of voluntary participating in the study and discontinuation as the patients see fit. The interview only proceeded with the agreement of participation by the patients. Oral consent was chosen due to the fact that the study involved minimal risk and it was very difficult to approach patients from various places in Vietnam.

### 2.2. Measure and Instruments

20-minute interview via telephone was conducted with each participant, in which a research questionnaire was used to collect information regarding patients' socioeconomic characteristics, disease characteristics, and HRQOL. The English version of the 15D questionnaire was translated into Vietnamese and validated by a crosscheck between our own research team. The translated version was then reviewed and adjusted (pronouns and some minor wording changes) for culture appropriation and lucidity. Lastly, a comparison between our translated version and the official version was made to ensure minimal differences. This process was done very carefully with assistance from various experts in the field. Researchers were trained for conducting research questionnaires and medical records procedures. The training process consisted of a minimum of 24 hours per researcher, mock interviews, and approval of the lead researcher.

Participants, who met the eligibility criteria, were contacted by the research team during the designated research timeline, which was carefully planned to ensure minimal impact on patients' daily lives. The purpose, the benefits, the drawbacks, and the confidentiality aspect of participating in the study were introduced to the participants when they were asked to join the study. Participants' consent was ensured prior to the interview.

The structured questionnaire was developed with the following information.

#### 2.2.1. Socioeconomic Characteristics

Participants were asked to self-report their information about gender, age, educational status, occupation, marital status, number of members in their family, monthly income, places where they lived, and their working time and number of working hours per day.

#### 2.2.2. Medical History of Participants or Participants' Family and Gastric Cancer Characteristics

Participants were asked for their medical history, including gastric disease-related history, time and place of their diagnosis, and treatment options that they had already undergone. Medical history of participants' family was also investigated.

#### 2.2.3. GC Characteristics in Participants' Medical Record

In addition to interviews, disease characteristics in participants' medical record, including gastric endoscopy, pathology results, diagnostic stages, and treatment therapies, were also collected.

Regarding the characteristics of GC, GC patients underwent gastroscopy to determine the location and size of the lesions. Patients also performed biopsy after surgery for histopathology sample for Lauren classification. Cancer stages were categorized according to the TNM system of the American Joint Committee on Cancer (T describes the size of the primary tumor and whether it has invaded nearby tissue, N describes regional lymph nodes that are involved, and M describes distant metastasis).

#### 2.2.4. Health-Related Quality of Life

The 15D is a widely used, validated, standardized, self-administered, generic instrument with 15 dimensions (including mobility, vision, hearing, breathing, sleeping, eating, speech, excretion, usual activities, mental function, discomfort and symptoms, depression, distress, vitality, and sexual activity) with five possible responses for each. Depending on the answers given by the respondents when they were asked about their state of health at the moment of the interview, a score between 1 and 5 (with 1 being the best value and 5 being the worst) would be chosen and for this estimation we used the Finnish valuation algorithm for utility scores and profiles. The evaluation system was based on an application of the multiattribute utility theory [7]. The single-index score (15D score), representing the overall HRQOL on a 0 to 1 scale (1=full health, 0=being dead) and the dimension level values, reflecting the goodness of the levels relative to no problem on the dimension (value=1) and to the state of being dead (value=0), is calculated from the health state descriptive system by using a set of population-based preference or utility weights. It is not possible to calculate the 15D score if more than three values are missing. The minimal clinically important difference (MID) in the 15D has been estimated at 0.03 [8]. The process of determining the utility score began with gathering data from 182 participants' answers. After that, we requested a conversion tool, which was a preprogrammed excel sheet with utility weights for each dimension and functions. The data was added onto the sheet, and the utility scores were automatically calculated.

We conducted a pilot survey of 20 participants of different ages, genders, and occupations, and only minor changes to the wording were made in order to meet participants' preferences and culture. These participants did not take part in the recruitment as well as in the conduct of the study.

### 2.3. Statistical Analysis

Data were analyzed using STATA 12.0 (Stata Corp. LP, College Station, TX, USA). Socioeconomic information, medical history, characteristic of gastric cancer disease, and quality of life of participants were described. These variables were considered potential covariates in the regression models. Because the measured domains were continuous variables, we applied multivariate Tobit regression to identify factors associated with quality of life of gastric cancer patients. We applied forward stepwise selection strategy to remove nonsignificant variables, and the threshold to select variables for reduced models was 0.2. A p-value of log likelihood ratio test less than 0.05 was considered as statistical significance. We also applied Psychometric properties: the instrument has Cronbach's alpha = 0.81, showing good internal consistency reliability.

### 2.4. Ethics Approval

This study was approved by the Ethics Council of Hanoi Medical University.

## 3. Results

Table 1 describes the socioeconomic characteristics of participants. There is no statistically significant difference in education between males and females (p=0.85). We found that 86.8% of the patients were living with a spouse (with a significant difference between males and female, p<0.01). The proportion of patients working as workers/farmers was 25.3%, followed by unemployment (20.9%), white collar (14.3%), and freelancer (13.7%). The mean age of the male group was higher than that of the female group (62.4 vs. 57.4 years; p=0.04).

Table 2 describes the disease characteristics of the participants. More than half of the patients (55.5%) had a history of gastric disease; there was no significant difference between females and males (p = 0.8). The majority of the patients had gastritis (66.3%). Time from diagnosis of gastric disease of more than 5 years was 38%, from 1–5 years was 39%, and less than 1 year was 23%. Patients with a family history of gastric cancer were 14.3 % (p=0.22).

Most patients (80.8%) were found with lesion size greater than 3 cm. Patients performed biopsy following surgery for histopathology sample. The intestinal type (according to Lauren classification) was found in 135 (75.4%) patients, three times higher than that of diffuse type (24.6%). The largest proportion of patients was on stages I and II (40%), males significantly more than females (p = 0.05). Regarding the treatment methods, surgery combined with chemotherapy was performed in 114 patients (62.6%), nearly triple the number of patients who only underwent surgical therapy (22%). The rest of the participants had other treatment methods, including radiation, palliative care, and traditional medicine. The estimated mean time from diagnosis was 14.8 ± 8.4 months.

**Figure 1:**
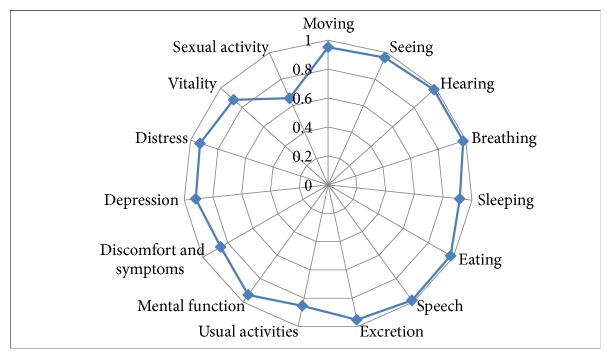
Quality of life of respondents on the 15D scale.

Figure 1 describes the quality of life in each dimension of the 15D scale. The average HRQOL score of GC patients assessed using the 15D instruments was 0.92 (standard of deviation = 0.08). Each dimension was represented as a point on the spider web figure scaling from 0 to 1. Most dimensions ranged between 0.86 and 0.95 with “sexual activity” having the lowest score of 0.66, followed by “usual activity,” “discomfort and symptoms,” and “vitality” scoring 0.85, 0.86, and 0.87, respectively. The other dimensions were all above 0.9.

Table 3 shows the multivariable regression model of HRQOL-related factors. HRQOL index was lower in patients with the educational level of at least high school (Coef. = -0.04, 95%CI = -0.07, -0.02) and being worker/farmer (Coef. = -0.04, 95%CI = -0.07, -0.01). Among the factors representing GC characteristics, there was a quality of life reduction in patients with stage III disease (Coef. = -0.04, 95%CI = -0.08, 0) and lymph nodes statuses N1 (Coef. = -0.04, 95%CI = -0.08, -0.01) and N2 (Coef. = -0.05, 95%CI = -0.10, 0.00) and in patients who did not receive surgical treatment (Coef. = -0.06, 95%CI = -0.10, -0.01).

Variables that are investigated but excluded in the final model are gender, marital status, religion, and patients' families' history of GC.

**Figure 2:**
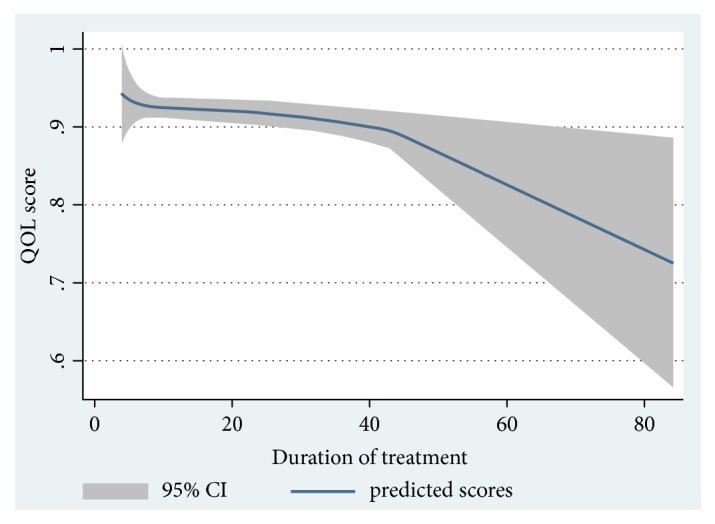
Association between quality of life and time from diagnosis.

Figure 2 demonstrates the statistically predicted inverse relationship between patients' average time from diagnosis and their quality of life, with the emphasis on the sharp decrease of HRQOL index starting from the 40^th^ month.

## 4. Discussion

The study found a relatively high HRQOL among gastric cancer patients. The dimension with the lowest score was “sexual activity.” Factors that affected GC patients' HRQOL were educational status, age, occupation, disease stage, treatment method, and time from diagnosis. This suggests the importance of therapeutic intervention with the goal to improve the GC patients' HRQOL on both clinical practice and healthcare management.

The average HRQOL score of GC patients discovered in this study was higher compared to HRQOL (measured by similar 15D instrument) of patients suffering from other types of cancer, in particular, lung cancer (0.80 ± 0.10) [9]; breast cancer during palliative care (0.72 ± 0.14) [10]; colorectal cancer (0.89 ± 0.10) [11]; prostate cancer (0.91 ± 0.09 during regional stage and 0.67 ± 0.10 during palliative care) [12]. This was possibly due to the fact that the majority of our participants were in their stable stages of the disease. This argument was favored by a study by Tyrvainen et al. using the 15D instrument on 25 long-term survivors after total gastrectomy, which observed that the quality of life of these patients was not significantly different from that of the normal population [13]. Among the dimensions measured, “sexual activity” had the lowest score. This was in line with current literature showing that one of the more frequent and serious adverse conditions of cancer and its treatment is sexual impairment [14, 15]. Disruption of sexual activity was found in both men and women [16]. A recent study on the sexual health of digestive cancer patients during chemotherapy has demonstrated the reduced frequency of sexual intercourse among postdiagnosis sexually active patients, with one-third of the study population being reported to have completely stopped sexual activity. Many of the respondents also expressed their desire for sexual care [17]. Therefore, improving “sexual activity” is necessary for gastric cancer patients.

The “usual activity” and “discomfort and symptoms” dimensions also scored lower compared to others, being 0.86 and 0.85, respectively. This indicates an impairment in the activities of daily living in GC patients. A significant number of patients reported experiencing difficulty in maintaining daily activities including employment, studying, housework, and free time activities after diagnosis and treatment. Physical activities were also found to be affected, when using the EORTC QLQ-C30 instrument to evaluate GC patients' quality of life, with the score of 69.4 ± 14.9 (for the group that got ECF (Epirubicin, cisplatin, and continuous 5-fluorouracil) chemotherapy) and 68.3 ± 19.1 (for the group that had TCF (Docetaxel, carboplatin, and 5-fluorouracil) chemotherapy); the instrument was scored from 0 to 100, with higher score showing better functioning [18, 19]. Many patients in our study mentioned postsurgery pain or numbness feeling of hands and feet after chemotherapy. Pain is also a common symptom among GC patients after treatment [18]. Thus, to improve rehabilitation, it is important for health providers to consult GC patients about the problems that they may have to confront after treatment.

According to our knowledge, researches on the topic of HRQOL in gastric cancer patients using the 15D instrument have been relatively limited. The majority of the current studies used EORTC QOL-C30, EORTC QLQ-STO22, and other instruments, focusing on different dimensions ranging from function, symptom, global health, and quality of life (EORTC QOL-C30) to other factors such as emotion (part of EORTC QLQ-STO22) [20–23]. Compared to those tools, the 15D instrument is more generic and simplistic. This can be considered an advantage due to the fact that it can be answered by more patients with different educational background in a shorter period of time. Furthermore, there are aspects not covered by other instruments, for example, “sexual activity.” Nevertheless, further studies utilizing a combination of generic and specific tools are needed in order to provide a more comprehensive assessment of patients' quality of life, which in turn would result in better intervention methods and improvement.

Patients' history of gastric disease has been considered one of the risk factors for gastric cancer. According to our research, two-thirds of GC patients had medical records of gastritis. In Vietnam, there has not yet been any national GC screening program which is as fully developed as in Japan [24], Korea [25], and China [26]. Nonetheless, the rate of patients having GC stages III and IV in Vietnam was found to be higher than that of Japan [27] and Korea [28]. The underdevelopment of the Vietnamese healthcare system combining with a generally found lack of knowledge from patients about the development of GC may contribute to the higher prevalence of GC in Vietnam. The establishment of early GC screening and systematic monitoring programs, as well as an improvement of public medical education, can be suggested as the new direction for future studies in Vietnam. Despite the fact that GC rates differ between nations, it is a common knowledge that family history is one of the main risk factors, with 2-3-fold higher rate presented in patients with a family history of cancer [29]. The percentage of patients that have a family history of GC in our research was 14.3%, which was similar to Italy (21.9%) [30] and Spain (17.6%) [31]. The high proportion of patients having surgery and chemotherapy corresponds with the current trend in GC treatment worldwide [32, 33]. The regression linear model for HRQOL showed lower scores in patients with higher education (above high school level) in comparison to that of patients with lower education (under high school). While some studies indicated no association between cancer patients' quality of life and their education, other studies had pointed to the opposite [34–36]. A possible reason for these findings is that highly educated patients tend to be able to do their own research regarding their conditions. The information they found may affect their quality of life negatively due to the pressure of having a disease. In addition, medical consultation for patients, especially those with cancers, is still a weak aspect of the Vietnamese healthcare system.

Patients' HRQOL during late stages (stage III in comparison to stage 0) and lymph node metastasis (statistically significant in N1 and N2 group compared to N0) were found to negatively correlate with quality of life among our participants. Patients with stage II or higher cancer reportedly had experienced decreased daily function and increased fatigue, pain, and loss of appetite. Furthermore, the condition worsens as the disease progresses; this leads to many complications that directly affect patients' quality of life, such as obstructions or bleeding due to ulcerations and lesions. This may explain the finding that the QOL scores of stage I cancer patients are higher than the overall GC QOL score [37]. Diffuse type GC patients (by Lauren classification) had a lower HRQOL score than those that have intestinal type. One possible reason for the result is that the diffuse carcinoma is more malignant than their intestinal counterpart due to the lower response rate to chemotherapy [38].

In our study, 15.4% of GC patients, who had other treatment options including radiation therapy, traditional medicine, or no treatment, had a lower HRQOL score compared to those who had surgical treatment or a combination of surgery and chemotherapy. Patients who underwent gastrectomy only were mostly early diagnosed with a lower degree of metastasis. The proportion of patients in the other groups (who chose traditional medicine), however, represents a predicament of the Vietnamese healthcare, in which cancer patients refuse to follow treatment plans from doctors and instead agree with traditional methods that are not yet proven to be effective by the scientific method. This may cost the patients the opportunity to get treatment in time and reduce their quality of life. Moreover, we found that GC patients' HRQOL decreased as the time from diagnosis increased, especially from the 40^th^ month onward. Research by Wang and colleagues also reported similar results when they showed the patients' HRQOL deteriorated over time [39]. Correct identification of the relationship between HRQOL index and the time from diagnosis, especially the point of time in which patients experience a significant decrease in quality of life (around the 40^th^ month as shown in this study), helps improve treatment strategy and intervention by clinicians.

### 4.1. Implications and Recommendation

Our findings denote potentials in enhancing therapeutic intervention as well as service management and medical policies aiming towards cancer patients. This would suggest the importance of providing systematic screening service for early detection of cancer, as well as frequently measuring personal functioning and performance of GC patients as parts of QOL assessment during clinical examination. Information from such assessments would help GC patients improve their quality of life through appropriate intervention and counseling. It also implies the needs for more focused policies on raising the overall quality of life of patients, such as encouragement of periodically HQROL assessment and acknowledging HRQOL as a treatment/intervention goal besides the 5-year survival rate.

### 4.2. Limitations

Due to the fact that the research was done on a relatively homogeneous patient group that came from the four central hospitals of Hanoi, the representativeness of the population was not high. Participants in this study could be representative of gastric cancer patients from the Northern region and from the same hospital level throughout Vietnam. This also led to the low statistical dispersion of the data. However, considering the novelty of the research in Vietnamese research literature, the selection of a more heterogeneous group was a challenge. International research on GC patients' HRQOL is currently limited; hence, the findings of our research still contribute to the overall scientific literature. Other drawbacks of our study include the lack of information regarding comorbidity and patients' family medical history, both of which can be made available in future research. These were mostly due to the limitation in the Vietnamese healthcare indicator system and lack of standards among hospitals, particularly in the management of documents and medical records. Nevertheless, such limitations can be improved in future study.

## 5. Conclusion

The 15D instrument was a suitable tool to assess Vietnamese gastric cancer patients' quality of life. “Sexual activity” having the lowest score indicates a huge issue in Vietnamese GC patients' HRQOL. This opens up research opportunities to improve the intervention and monitoring program regarding GC patients' HRQOL. The HRQOL has also been found to be negatively associated with education background, age, more severe stages of disease, and longer time from diagnosis.

## Figures and Tables

**Table 1:** Characteristics of respondents.

	Male		Female		Total		P
	N	%	N	%	N	%	
*Education*							
< High School	73	58.9	35	60.3	108	59.3	0.85
>= High School	51	41.1	23	39.7	74	40.7	
*Marital status*							
Living with spouse/partner	117	94.4	41	70.7	158	86.8	<0.01
Others	7	5.7	17	29.3	24	13.2	
*Occupation *							
Unemployment	20	16.1	18	31.0	38	20.9	0.20
Freelancer	18	14.5	7	12.1	25	13.7	
White collar	20	16.1	6	10.3	26	14.3	
Worker/Farmer	34	27.4	12	20.7	46	25.3	
Others	32	25.8	15	25.9	47	25.8	

	*Mean*	*SD*	*Mean*	*SD*	*Mean*	*SD*	

*Age*	62.4	9.9	57.4	14.1	60.8	11.6	0.04

**Table 2:** Disease characteristics of participants according to genders.

	Male		Female		Total		P
	n	%	n	%	n	%	
*History of Gastric diseases *	68	54.8	33	56.9	101	55.5	0.80
*Diagnosing gastric diseases *							
Gastritis	44	67.7	21	63.6	65	66.3	0.67
Others	21	32.3	12	36.4	33	33.7	
*Time to diagnosis *							
> 5 years	28	41.2	10	31.3	38	38.0	0.30
1-5 years	23	33.8	16	50.0	39	39.0	
< 1 year	17	25.0	6	18.8	23	23.0	
*Family history with gastric cancer*	15	12.1	11	19.0	26	14.3	0.22
*Characteristics of Gastric cancer Lesion size*							
<= 3 cm	19	16.4	14	25.0	33	19.2	0.18
> 3 cm	97	83.6	42	75.0	139	80.8	
*Lauren Classification*							
Intestinal type	95	77.9	40	70.2	135	75.4	0.27
Diffuse type	27	22.1	17	29.8	44	24.6	
*Tumor Size*							
T1 and T2	9	10.3	6	17.7	15	12.4	0.10
T3	40	46.0	20	58.8	60	49.6	
T4	38	43.7	8	23.5	46	38.0	
*Node*							
N0	16	19.1	4	11.4	20	16.8	0.70
N1	39	46.4	16	45.7	55	46.2	
N2	20	23.8	11	31.4	31	26.1	
N3	9	10.7	4	11.4	13	10.9	
*Stage*							
I and II	29	32.6	11	32.4	40	32.5	0.05
III	33	37.1	5	14.7	38	30.9	
IV	3	3.4	3	8.8	6	4.9	
Not defined	24	27.0	15	44.1	39	31.7	
*Treatment*							
Surgery	28	22.6	12	20.7	40	22.0	0.96
Chemotherapy + surgery	77	62.1	37	63.8	114	62.6	
Others	19	15.3	9	15.5	28	15.4	
*Time from diagnosis*	14.2	6.8	16.3	11.1	14.8	8.4	0.30

**Table 3:** Associated factors related to the quality of life.

	Coef.	p	95%CI	
*Education (vs High school)*				
>= High School	-0.04*∗*	<0.01	-0.07	-0.02
*Occupation (vs Unemployment)*				
Worker, farmer	-0.04*∗*	0.01	-0.07	-0.01
*Gastric diseases (vs Gastritis)*				
Other (atrophic gastritis)	0.02	0.23	-0.01	0.05
*Stage (vs I)*				
III	-0.04*∗*	0.03	-0.08	0.00
*Lauren Classification (vs Intestinal type)*				
Diffuse type	-0.02	0.33	-0.05	0.02
*Treatment (vs surgery)*				
Others	-0.06*∗*	0.01	-0.10	-0.01
*Lymph node (vs N0)*				
N1	-0.04*∗*	0.02	-0.08	-0.01
N2	-0.05*∗*	0.05	-0.10	0.00
N3	0.04	0.16	-0.02	0.10
*Age*	-0.001	0.09	-0.003	0.0002

*∗p* < 0.05

Note: The reference categories consist of dependent variables (in the parenthesis of the related variables), namely, High school (in Education), unemployment (in occupation), gastritis (in Gastric disease). Stage I (in Stage), Intestinal type (in Lauren classification), surgery (in Treatment), Lymph node N0 (in Lymph Node).

## Data Availability

The data sets generated during and/or analyzed during the current study are available from the corresponding author on reasonable requests. Requests for access to these data should be made to Van Thanh Ta, Biochemistry Department, Hanoi Medical University, Hanoi, Vietnam; Email: tathanhvan@hmu.edu.vn.

## References

[1] Bray F., Ferlay J., Soerjomataram I. (2018). Global cancer statistics 2018: GLOBOCAN estimates of incidence and mortality worldwide for 36 cancers in 185 countries. *CA: A Cancer Journal for Clinicians*.

[2] Binh T. T., Tuan V. P., Dung H. D. (2017). Advanced non-cardia gastric cancer and Helicobacter pylori infection in Vietnam. *Gut Pathogens*.

[3] Tran T. V., Anh P. T., Huong T. T. T. (2017). *Cancer Control in Vietnam: Where are we?*.

[4] Zali H., Rezaei-Tavirani M., Azodi M. (2011). Gastric cancer: prevention, risk factors and treatment. *Gastroenterology and Hepatology from Bed to Bench*.

[5] Ngoan L. T., Lua N. T., Hang L. T. M. (2007). Cancer mortality pattern in Viet Nam. *Asian Pacific Journal of Cancer Prevention*.

[6] Osoba D. (2011). Health-related quality of life and cancer clinical trials. *Therapeutic Advances in Medical Oncology*.

[7] Richardson J., Iezzi A., Khan M. A. (2015). Why do multi-attribute utility instruments produce different utilities: the relative importance of the descriptive systems, scale and ‘micro-utility’ effects. *Quality of Life Research*.

[8] Sintonen H. (2001). The 15D instrument of health-related quality of life: properties and applications. *Annals of Medicine*.

[9] Ilonen I. K., Räsänen J. V., Sihvo E. I. (2007). Pneumonectomy: post-operative quality of life and lung function. *Lung Cancer*.

[10] Färkkilä N., Torvinen S., Roine R. P. (2014). Health-related quality of life among breast, prostate, and colorectal cancer patients with end-stage disease. *Quality of Life Research*.

[11] Färkkilä N., Sintonen H., Saarto T. (2013). Health-related quality of life in colorectal cancer. *Colorectal Disease*.

[12] Torvinen S., Färkkilä N., Sintonen H., Saarto T., Roine R. P., Taari K. (2013). Health-related quality of life in prostate cancer. *Acta Oncologica*.

[13] Tyrväinen T., Sand J., Sintonen H., Nordback I. (2008). Quality of life in the long-term survivors after total gastrectomy for gastric carcinoma. *Journal of Surgical Oncology*.

[14] Schover L. R., van der Kaaij M., van Dorst E., Creutzberg C., Huyghe E., Kiserud C. E. (2014). Sexual dysfunction and infertility as late effects of cancer treatment. *European Journal of Cancer Supplements*.

[15] Sadovsky R., Basson R., Krychman M. (2010). Cancer and sexual problems. *The Journal of Sexual Medicine*.

[16] Strasser F., Palmer J. L., Schover L. R. (2006). The impact of hypogonadism and autonomic dysfunction on fatigue, emotional function, and sexual desire in male patients with advanced cancer. *Cancer*.

[17] Almont T., Couteau C., Etienne H. (2018). Sexual health and needs for sexology care in digestive cancer patients undergoing chemotherapy: a 4-month cross-sectional study in a French University Hospital. *Supportive Care in Cancer*.

[18] Sadighi S., Mohagheghi M. A., Montazeri A., Sadighi Z. (2006). Quality of life in patients with advanced gastric cancer: a randomized trial comparing docetaxel, cisplatin, 5-FU (TCF) with epirubicin, cisplatin, 5-FU (ECF). *BMC Cancer*.

[19] Park S. H., Lee W. K., Chung M. (2006). Quality of life in patients with advanced gastric cancer treated with second-line chemotherapy. *Cancer Chemotherapy and Pharmacology*.

[20] McCall M. D., Graham P. J., Bathe O. F. (2016). Quality of life: A critical outcome for all surgical treatments of gastric cancer. *World Journal of Gastroenterology*.

[21] Eguchi T., Fujii M., Wakabayashi K. (2003). Docetaxel plus 5-fluorouracil for terminal gastric cancer patients with peritoneal dissemination. *Hepato-Gastroenterology*.

[22] Aaronson N. K., Ahinedzai S., Bergman B. (1993). The European Organization for Research and Treatment of Cancer QLQ-C30: a quality-of-life instrument for use in international clinical trials in oncology. *Journal of the National Cancer Institute*.

[23] Blazeby J. M., Conroy T., Bottomley A. (2004). Clinical and psychometric validation of a questionnaire module, the EORTC QLQ-STO 22, to assess quality of life in patients with gastric cancer. *European Journal of Cancer*.

[24] Tashiro A., Sano M., Kinameri K., Fujita K., Takeuchi Y. (2006). Comparing mass screening techniques for gastric cancer in Japan. *World Journal of Gastroenterology*.

[25] Lee K. S., Oh D. K., Han M. A. (2011). Gastric cancer screening in Korea: report on the national cancer screening program in 2008. *Cancer Research and Treatment*.

[26] Riecken B., Pfeiffer R., Ma J. L. (2002). No impact of repeated endoscopic screens on gastric cancer mortality in a prospectively followed Chinese population at high risk. *Preventive Medicine*.

[27] Inoue M., Tsugane S. (2005). Epidemiology of gastric cancer in Japan. *Postgraduate Medical Journal*.

[28] Choi K. S., Jun J. K., Suh M. (2015). Effect of endoscopy screening on stage at gastric cancer diagnosis: results of the National Cancer Screening Programme in Korea. *British Journal of Cancer*.

[29] Rokkas T., Sechopoulos P., Pistiolas D., Margantinis G., Koukoulis G. (2010). Helicobacter pylori infection and gastric histology in first-degree relatives of gastric cancer patients: a meta-analysis. *European Journal of Gastroenterology & Hepatology*.

[30] Bernini M., Barbi S., Roviello F. (2006). Family history of gastric cancer: A correlation between epidemiologic findings and clinical data. *Gastric Cancer*.

[31] García-González M. A., Lanas A., Quintero E. (2007). Gastric cancer susceptibility is not linked to pro-and anti-inflammatory cytokine gene polymorphisms in whites: a nationwide multicenter study in Spain. *American Journal of Gastroenterology*.

[32] Paoletti X., Oba K., Burzykowski T. (2010). Benefit of adjuvant chemotherapy for resectable gastric cancer: a meta-analysis. *Journal of the American Medical Association*.

[33] Macdonald J. S., Smalley S. R., Benedetti J. (2001). Chemoradiotherapy after surgery compared with surgery alone for adenocarcinoma of the stomach or gastroesophageal junction. *The New England Journal of Medicine*.

[34] Heydarnejad M. S., Hassanpour Dehkordi A., Solati Dehkordi K. (2011). Factors affecting quality of life in cancer patients undergoing chemotherapy. *African Health Sciences*.

[35] Dehkordi A., Heydarnejad M. S., Fatehi D. (2009). Quality of life in cancer patients undergoing chemotherapy. *Oman Medical Journal*.

[36] Wang Y., Wang H., Yin G., Tian H., Wang D. (2010). The quality of life of Chinese middle-aged male patients with gastric carcinoma after total gastrectomy and nursing intervention. *Clinical Oncology and Cancer Research*.

[37] Suk H., Kwon O. K., Yu W. (2015). Preoperative quality of life in patients with gastric cancer. *Gastric Cancer*.

[38] Petrelli F., Berenato R., Turati L. (2017). Prognostic value of diffuse versus intestinal histotype in patients with gastric cancer: A systematic review and meta-analysis. *Journal of Gastrointestinal Oncology*.

[39] Wang S.-Y., Hsu S. H., Gross C. P. (2016). Association between time since cancer diagnosis and health-related quality of life: a population-level analysis. *Value in Health*.

